# TGFβ Superfamily Members as Regulators of B Cell Development and Function—Implications for Autoimmunity

**DOI:** 10.3390/ijms19123928

**Published:** 2018-12-07

**Authors:** Esther Tamayo, Pilar Alvarez, Ramón Merino

**Affiliations:** 1 Departamento de Biología Molecular-IDIVAL Universidad de Cantabria, Santander 39011, Spain; 2 Instituto de Biomedicina y Biotecnología de Cantabria, Consejo Superior de Investigaciones Científicas-Universidad de Cantabria, Santander 39011, Spain; pilarasmaza@gmail.com

**Keywords:** TGFβ superfamily, TGFβ signaling, B cell, immune homeostasis, tolerance, autoimmunity

## Abstract

The TGFβ superfamily is composed of more than 33 growth and differentiation factors, including TGFβ1, β2, β3, BMPs, GDFs, nodal-related proteins, and activins. These members usually exert pleiotropic actions on several tissues and control multiple cellular processes, such as cell growth, cell survival, cell migration, cell fate specification, and differentiation, both during embryonic development and postnatal life. Although the effects of these factors on immune responses were elucidated long ago, most studies have been focused on the actions of TGFβs on T cells, as major regulators of adaptive immunity. In this review, we discuss new findings about the involvement of TGFβ superfamily members in the control of B cell development and function. Moreover, the potential contribution of TGFβ signaling to control B cell-mediated autoimmune diseases and its utility in the design of new therapies are also discussed.

## 1. Introduction

The transforming growth factor beta (TGFβ) superfamily comprises a large family of growth and differentiation factors (to date, more than 33 members have been identified in humans), which act mainly as morphogens and/or cytokines. These factors play multiple functions during embryonic development and postnatal life [1,2]. In the immune system, the TGFβ superfamily member most extensively studied is TGFβ1, which plays a pivotal role in the control of immune homeostasis and inflammation [3,4]. The involvement of other family members has also been revealed more recently. Most of these studies have been focused on T cells as central effectors and regulators of adaptive immune response and tolerance [5,6,7]. However, TGFβ superfamily members also regulate B cells, which exert crucial roles in immunity through both antibody-independent and antibody-dependent activities. This review describes the different actions of TGFβ superfamily members on B cells from their development in bone marrow to their maturation into antibody-secreting plasma cells at peripheral lymphoid tissues, and their involvement in the control of self-tolerance and autoimmunity. Consequently, the modulation of B cell activity by the TGFβ superfamily could contribute to the development of new therapeutic strategies for autoimmune diseases.

Moreover, in recent years, the microbiota has emerged as a master regulator of immune homeostasis [8]. Alterations in the normal composition of commensal bacteria in the gut, termed as dysbiosis, are associated with a wide range of inflammatory and/or autoimmune pathologies [9]. TGFβ contributes to maintaining the gut microbiota and immune tolerance-promoting IgA (Immunoglobulin A) production by B cells, which in turn constitutes a barrier for the adhesion, growth, and pass of potential pathogens and their harmful derivatives [8].

### TGFβ Superfamily Members and Signaling 

As mentioned above, the TGFβ superfamily comprises a vast variety of members displaying pleiotropic autocrine, paracrine, or endocrine activities. These growth and differentiation factors are divided into two functional groups: the TGFβ type group, which includes TGFβs, activins, nodal and some growth and differentiation factors (GDFs); the bone morphogenetic protein (BMP) type group, which includes BMPs, most GDFs, and anti-Müllerian hormone (AMH). Although the different TGFβs, BMPs, and activins share components across the TGFβ signaling pathway, each step within it is controlled by a repertory of cell extrinsic and intrinsic regulators at multiple levels (ligand bioavailability, receptor complex formation, positive and negative intermediaries), resulting in functional specificity and diversity of each member. The accumulated evidence from the last 40 years has shown that TGFβ signaling effects are both cell type and context dependent [1,10,11,12].

**Figure 1:**
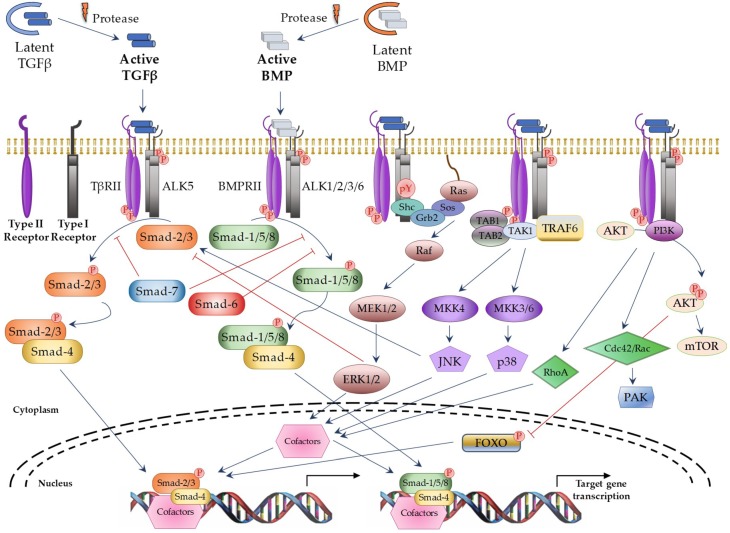
TGFβ superfamily signaling through canonical and non-canonical pathways. Blue arrows: activation; red ―|: inhibition; red circle with P in it: phosphorylation; dotted curve: nuclear membrane; black arrows: induction of gene transcription. TAB1/2: TGFβ Activated Kinase 1 Binding Protein 1 and 2; TAK1: TGFβ Activated Kinase 1; TRAF6: TNF receptor associated factor 6; AKT: protein kinase B; MEK: Mitogen-activated protein kinase kinase; MKK: Mitogen-activated protein kinase kinase; mTOR: Mammalian target of rapamycin; PAK: p21 activated kinase; FOXO: Forkhead box O.

TGFβ superfamily members signals through a heterotetrameric receptor complex composed of two type I (TβRI; also known as ALKs: activin receptor-like kinases) and two type II (TβRII) transmembrane receptor subunits with serine/threonine kinase domains. Seven TβRI and five TβRII have been identified, which may form specific heterocomplexes for each TGFβ superfamily member (Table 1). After ligand binding to the TβRII subunits, the TβRI subunits are recruited to form the activated complex receptor and then, the signal can be transduced into the nucleus through different pathways. The “canonical” or “Smad-dependent” routes are mediated by a family of evolutionary highly conserved proteins, known as Smads (small mothers against decapentaplegic) (Figure 1 on the left). In vertebrates, eight different Smad proteins have been described, that are divided into three different types according to their function: receptor-regulated Smads (R-Smads: Smad-1, -2, -3, -5, and -8), common-mediator Smad (Co-Smad: Smad-4), and inhibitory Smads (I-Smads: Smad-6 and -7). R-Smads are phosphorylated by specific activated TβRIs. Thus, Smad-2 and -3 are activated by TGFβ and Activin receptors, whereas Smad-1, -5, and -8 are activated by BMP receptors [10]. Following complex receptor formation, TβRI is phosphorylated by TβRII and it then recruits and phosphorylates R-Smads. Activated R-Smad proteins associate with Smad4 and translocate into the nucleus, where they recruit additional transcriptional regulators, including DNA-binding transcription factors, co-activators, co-repressors, and chromatin remodeling factors, that control the expression of numerous target genes. In addition, TGFβ can activate several “Smad-independent signaling pathways” [2,13], including ERK (extracellular signal–regulated kinase), JNK (c-Jun N-terminal kinase), p38, RhoA, PI3K (Phosphatidylinositol 3-kinase), and AKT (protein kinase B) in a cell type-specific and context-dependent manner (Figure 1 on the right). Activation of these pathways may also contribute to the cellular responses induced by TGFβ.

Therefore, the TGFβ superfamily signaling pathway is a highly complex process that is finely controlled by positive and negative regulators to ensure its physiological functions in different cells and tissues.

In the immune system, TGFβ1 has been the superfamily member most extensively studied. More recently, other TGFβ superfamily members have also been involved in the regulation of this system. Multiple immune cell types express TGFβ superfamily ligands and/or receptors and respond to these growth factors in an autocrine, paracrine, or even endocrine manner. The effects of TGFβs are cell type and maturation stage-dependent. These growth and differentiation factors possess effects in both the innate and adaptive immune system [6,7]. This review focusses on the reported direct effects for TGFβ superfamily members on B cells. However, it is important to point out that B cell response can also result indirectly affected through effects on other immune innate cells and T lymphocytes, which interact with B cells.

To summarize, after stimulation by infection, injury, or vaccination, dendritic cells (DCs) positively regulate MHC-II (Major Histocompatibility Complex class II) and costimulatory molecules and increase the production of cytokines. It licenses them to induce activation and differentiation of T cells [14]. Addition of TGFβ1 to DCs stimulated by lipopolysaccharide (LPS), TNF-α (Tumor Necrosis Factor α), and IL-1β (Interleukin 1β) inhibits the expression of MHC-II and costimulatory molecules and prevents the production of IL-12 [15]. Furthermore, mice with a deficiency in TβRII specifically in DCs (Tgfbr2^f/f^ Cd11c-Cre) develop a spontaneous multi-organ inflammation at 15 weeks of age, further emphasizing the importance of TGFβ signaling in DCs to control T and B cell activation [16]; BMPs favor DC activation and differentiation. In this regard, BMP-7 drives Langerhans cell differentiation [17]; DCs produce Activin A that inhibits their own maturation and their ability to produce inflammatory cytokines, affecting also T cell proliferation [18,19]. 

TGFβ1 is a potent chemoattractant factor of monocytes, fibroblasts, neutrophils, and mast cells [20,21], participating also in the functional polarization of macrophages and neutrophils from an inflammatory type I phenotype (displaying phagocytic actions) towards a type II phenotype (with reduced effector functions but producing large amounts of cytokines such as IL-6, IL-11 and TGFβ) [22]. A dual role for BMPs has been observed in macrophages. BMP-6 signaling, through BMPRII (Bone Morphogenetic Protein type II Receptor) and ALK-2, positively regulates IL-6 production, whereas signaling through ActRIIB inhibits it. In addition, BMP-6 induces iNOS (Inducible Nitric Oxide Synthase) and TNF-α production but inhibits macrophage growth [23,24]. Activin A is also secreted by macrophages in response to either pro-inflammatory or anti-inflammatory stimuli. This growth factor has adaptable effects in macrophage function, acting as a pro-inflammatory cytokine at steady state and as an anti-inflammatory agent under activation conditions [25,26].

Regarding T cells, mice with a selective deficiency or impairment of TGFβ signaling in T cells develop a severe and lethal autoimmune syndrome, stressing the importance of TGFβ activity in the regulation of T-cell activity [27,28]. TGFβ1 is potent inhibitor of T cell proliferation [29,30] and also affects cell survival. In some contexts, it promotes cell death to limit the expansion of T lymphocytes after activation [31,32]. However, in other circumstances, TGFβ1 promotes the survival of activated T cells by blocking FasL-mediated apoptosis [33]. Early differentiation of thymus-derived FoxP3^+^ regulatory T cells (Tregs) requires TGFβ signaling that improves the survival of these cells [34,35]. Furthermore, TGFβ1 also plays a central role in the differentiation of naïve CD4^+^ T cells into different effector subsets and allows plasticity between different CD4^+^ T responses. TGFβ1 promotes differentiation of both Foxp3+ regulatory T cells (Tregs) and T helper type 17 cells (Th17) depending on the absence or presence of pro-inflammatory cytokines, respectively [36,37,38,39], whereas suppresses the differentiation of T helper type 1 (Th1) and T helper type 2 cells (Th2) [40,41]. In addition, TGFβ1 cooperates with IL-4 to promote the differentiation of Th9 cells, which mediate defense against helminth infections and tumors, and allergic responses [42]. Likewise, as described in the Section 2.3, TGFβ influences the differentiation of follicular T helper cells (Tfh), responsible for T-dependent humoral responses. TGFβ1 also suppresses CD8^+^ T cell effector functions through the inhibition of perforin and IFNγ (Interferon γ) expression [27]. Moreover, it has been observed that developing Th17 cells, when exposed to IL-23, are able to produce TGFβ3 and become pathogenic Th17 cells [43]. On the other hand, in mature CD4^+^ T cells, inhibition of BMP signaling leads to the induction and suppression of Treg and Th17 differentiation, respectively [44]. Activin A favors the differentiation towards Th9 cells in a similar way than TGFβ1 [45], and synergizes with it during the differentiation of Tregs [46].

## 2. TGFβ Superfamily in B Cells

### 2.1. B Cell Development

**Figure 2:**
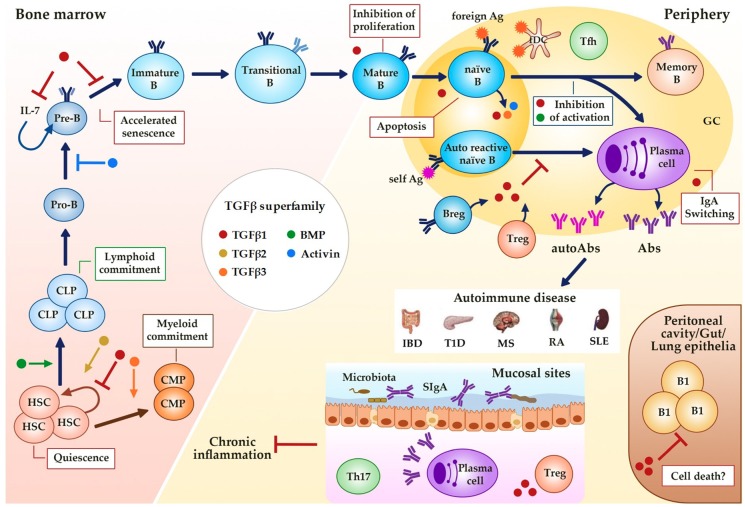
Regulation of B cell development and activity by TGFβ superfamily members. TGFβs, BMPs and activins affect hematopoiesis (quiescence and linage commitment in HSCs) and B cell development (maintenance and senescence of Pre-B cells) in the bone marrow, and immune function at the periphery (proliferation, survival, Ig class switching). Alterations in this regulation could result in different B cell-associated autoimmune diseases. GC (Germinal Center), HSC (Hematopoietic Stem Cell), CLP (Common Lymphoid Progenitor), CMP (Common Myeloid Progenitor), B reg (regulatory B cell), Treg (regulatory T cell), fDC (follicular dendritic cell), Tfh (follicular helper T cell), Ag (Antigen), Abs (Antibodies), SIgA (secretory IgA), IBD (Inflammatory Bowel Disease), T1D (Type 1 Diabetes), MS (Multiple Sclerosis), RA (Rheumatoid Arthritis), SLE (Systemic Lupus Erythematosus). Dark blue arrows show the sequence of B cell precursors during their differentiation in the bone marrow of after the activation of mature B cells in the periphery. The blue arrow, the yellow arrow, the green arrow, the orange arrow, the brown arrow and the red-brown arrow mean activation by the indicated (see color) TGFβ superfamily member. The red ―| and the blue ―| mean inhibition by the indicated (see color) TGFβ superfamily member. Red boxes indicate the activities induced by TGFβ superfamily member indicated by the color of the adjacent dot.

The TGFβ superfamily plays a crucial role in maintaining the self-renewal capacity of normal hematopoietic stem cells (HSCs) and in determining cell fate and hematopoietic lineage selection (Figure 2). In fact, active sites of hematopoiesis such as fetal liver and bone marrow are known to have abundant presence of TGFβ [47,48]. TGFβ signaling is a major regulator of HSCs function in vivo, with TGFβ1 and TGFβ3 acting as potent negative regulators and TGFβ2 as a positive regulator of the HSC population (reviewed in [49]).

Studies in mice with a conditional ablation of the BMP signaling pathway indicate that they are dispensable for intrinsic HSC maintenance in vivo, but this pathway is involved in HSC fate determination [50,51]. Thus, TGFβ1 specifically supports My-HSC (myeloid linage progenitors) proliferation [52], whereas BMP activation preferentially sustains lymphoid commitment of adult HSCs [51].

TGFβ1 is a potent regulator of B cell development from the pre-B cell stage up to immunoglobulin-secreting plasma cells. Light chain rearrangement and expression drive the transition from pre-B cells to immature B cells. In this regard, TGFβ1 inhibits κ light chain expression in murine pre-B cell clones [53] and exerts a selective inhibitory effect on the acquisition of cell surface λ light chains during the in vitro differentiation of normal human pre-B cells [54]. Moreover, TGFβ1 causes a dose-dependent impairment of lymphoid progenitor proliferation secondary to the inhibition of stromal IL-7 secretion [55]. 

Like TGFβ, BMPs and in particular BMP-6 has a negative effect in B lymphopoiesis, inhibiting cell proliferation through the upregulation of Id1 and Id3, two important Smad targets in early B cell differentiation [56]. Activins have also been involved in several processes of the B cell biology. Several experiments using murine or human cells show that stromal Activin A in bone marrow exerts inhibitory effects at early stages of B cell development under steady state conditions, acting as a brake signal on B cell progenitors controlling cell cycle arrest and apoptosis. However, in the presence of inflammatory signals (i.e., LPS (Lipopolysaccharide)), the bone marrow expression of Activin A turns off, allowing B cell differentiation and expansion [57,58,59].

### 2.2. B Cell Proliferation and Survival

The maintenance of homeostasis and tolerance in the immune system relays on the fine-tuning of cell activation, proliferation, and death. It is well known that a variety of cell types suffer cell-cycle arrest after in vitro exposure to TGFβ1 or TGFβ2 [53,60,61,62]. The anti-proliferative effects of TGFβ appear to be secondary to the inhibition in the expression of different transcription factors that induce cell growth, such as c-myc and the cell differentiation inhibitory Id proteins, resulting in enhanced expression of CDK (Cyclin-dependent kinase) inhibitors (reviewed in [63]). In both murine and human B cells, exogenous TGFβ inhibits the in vitro proliferation induced by different stimuli such as mitogens, CD40 or membrane IgM (Immunoglobulin M) ligation [61,64,65,66]. The inhibition of cell growth in these B cells is associated with G0/G1 arrest probably induced by cyclin A, c-myc repression, CDK2 inactivation, and expression of p21Cip1 and p27kip1 [67,68,69]. Interestingly, the same stimuli also promote the production of TGFβ in mature B cells as a probable negative feedback mechanism controlling excessive proliferative responses [61,64,65]. Activated mature human B cells also upregulate BMP-6 after BCR (B-cell receptor) stimulation, and exogenous BMP-6 inhibits their growth in association with Id1 upregulation [70]. An intriguing observation is that B cells from mice deficient in the BMP antagonist Twisted gastrulation (TWSG1) exhibit intrinsic defects that increase their proliferative responses in vitro. Although mature B cells from these deficient mice show a genetic profile compatible with a BMP imprinting, their altered in vitro proliferative responses are not modulated either through addition of recombinant BMP, other BMP antagonists, or recombinant TWSG1 itself, suggesting that TWSG1 alters B cell responses in an early maturation or selection steps [71].

In addition to the inhibition of B cell proliferation, TGFβ possesses strong pro-apoptotic effect in these cells both at the level of B cell precursors in the bone marrow and in peripheral mature cells. TGFβ1 triggers in vitro apoptosis both in normal B cells and in some transformed B cell lines, inducing pro-apoptotic members of the Bcl-2-family (such as Bim and BIK (BCL2 interacting killer)) while downregulating anti-apoptotic members such as Bcl-xL (B-cell lymphoma-extra large) [72,73,74,75]. Furthermore, mice with a selective B cell deficiency of TβRII (Tgfbr2^f/f^ Cd19-Cre, here named B-TβRII-KO) show decreased in vivo life-span of splenic non-cycling and long-living conventional B cells (measured by turnover from BrdU (5-Bromo-2’-deoxyuridine) labelling of B cell progenitors), when compared to wild type B cells. However, peritoneal B1 cells (self-renewal population) from the same mutant mice do not show an increased BrdU incorporation, but accumulate in these animals [76]. It suggests that TGFβ may have both pro-apoptotic and anti-apoptotic effects in B cells that are cell-context and/or cell-development dependent. Recently it has been reported that BMP-7 also induces apoptosis in human germinal center (GC) B cells, which, in opposition to naïve B cells, expressed high levels of BMP type I receptor but low levels of type II receptors [77]. 

TGFβ superfamily signaling links with numerous pathways (PI3K/Akt, c-myc (avian myelocytomatosis virus oncogene cellular homolog), JNK/p38, extrinsic or intrinsic apoptosis, etc.) regulating cell-death and survival, therefore the cell’s choice between proliferation, cell-cycle arrest, and apoptosis requires an integrated interpretation of multiple signaling inputs (i.e., from BCR, TLRs (Toll-like Receptors), CD40, FcRs (Immunoglobulin fragment crystallizable region receptor), cytokine receptors, metabolites, etc.). It is in turn dependent on the epigenetic and transcriptional state of each individual cell.

### 2.3. Activation, Immunoglobulin (Ig) Production and Differentiation

The development of an adaptive humoral response requires that naïve mature B cells undergo somatic hypermutation (SHM) and class switch recombination (CSR) upon antigen recognition through BCR, allowing them to differentiate into either memory B cells or immunoglobulin (Ig)-secreting plasma cells. These events lead to change in BCR affinity and isotype switching (from IgM to IgG, IgA, or IgE) that are regulated by TLR signaling, BCR coreceptors (i.e., CD19), enzymes (i.e., AID (activation-induced deaminase)), and cytokines. Genetic alterations, besides environmental factors (as described in the last section of this review) could alter that processes, thus giving rise to high-affinity autoreactive B cells, autoantibody production, and tissue damage, finally resulting in humoral autoimmune diseases [78].

Early findings showed that exogenous TGFβ1 decreased Ig secretion in human B lymphocyte stimulated in vitro with *Staphylococcus aureus* and IL-2, by inhibiting the synthesis of Ig mRNA and the switch from membrane-bound to secreted Ig forms [79]. In this regard, TGFβ signaling induced the expression of BCR signaling inhibitors (CD72, SHIP-1) and reduced the activation of the Jak/Stat pathway promoted by IL-4 [80]. However, a subsequent study reported that at low doses, TGFβ1 promoted, but high doses inhibited, antibody secretion in LPS-stimulated murine B cells [81]. Shortly afterwards, several groups evidenced that TGFβ signaling induced α germ-line Ig transcripts both in mouse and human uncommitted IgM^+^ B cells by activating Smad-2 and -3, which then associated with Smad-4 and Runx3 in the regulatory region of the Cα gene (reviewed in [82]). The biological significance of these in vitro studies was confirmed in vivo by using B-TβRII-KO mice, which exhibited B cell hyperresponsiveness, expansion of the B1 cell population, enhanced antibody production after immunization with weak antigens, a selective defect in IgA production and an increased anti-DNA autoantibody production [76]. Partially opposed effects were reported in mice with reduced Smad-7 activity, where a hyperactive TGFβ signal in B cells showed increased Ig class switching to IgA and enhanced spontaneous B-cell apoptosis, with reduced proliferative response to LPS stimulation [83]. The TGFβ signaling pathways responsible for the B-cell alterations that appeared in these mutant mice remained to be fully elucidated. In this regard, it was reported that mice with Smad-2 deficiency selectively in B cells (B-Smad2-KO), failed to develop B cell hyperresponsiveness and had normal follicular B cell numbers in the spleen but reduced marginal zone B cells. In contrast, they showed expanded peritoneal B1a cells and conventional B cells in Peyer’s patches, indicative of a different requirement of TGFβ for B cell homeostasis in different locations. Moreover, while serum levels of IgA were only mildly reduced, antigen-specific IgA antibody responses were strongly blocked. Unlike B-TβRII-KO mice, antigen-specific antibody responses of the rest of Ig isotypes and the B cell proliferative responses were normal in these B-Smad2-KO mice [84]. An interesting observation was the impairment of antibody immune responses in mice with a B cell-specific deficiency in TGFβ activated kinase 1 (TAK1) [85], one of the kinases activated during the Smad-independent TGFβ signaling pathways [86]. Since TAK1 was also involved in cellular responses to TLR ligands, CD40, and B cell receptor crosslinking [85], the relevance of these findings in the context of TGFβ activities on B cells remains to be elucidated. However, all these studies illustrate the complexity of TGFβ biology on B cell activation that results from the still poorly understood process of interactions/compensations between the different TGFβ signaling pathways.

BMPs also influence B cell function. Human naïve and memory B cells express type I and type II BMP receptors and different BMPs (BMP-2, -4, -6, or -7) block the in vitro anti-CD40/IL-2-induced production of IgM, IgG, and IgA, but with some particularities. BMP-7 inhibits DNA synthesis and induces apoptosis, counteracting the viability-promoting activity of anti-CD40. In turn, BMP-6 inhibits plasmablast differentiation [87]. 

Activins have been found to act in a similar way to TGFβ1, partially because these molecules share the Smad-2 and Smad-3 signaling intermediates. However, their effects on B cells seem to be stage development dependent and quite different to that of TGFβ. Activin A can induce cell cycle arrest and apoptosis at early stages of B cell development in the bone marrow [58]. LPS in vitro stimulation or antigen immunization in mice induces B cells to produce high amounts of activin-βA subunit as well as to reduce the secretion of its inhibitor follistatin. Furthermore, mature B cells express type I and type II activin receptors, suggesting that they are targets of activin. Pretreatment of naïve B cells with activin A before, but not at the same time as LPS activation resulted in increased cell growth and IgG production, indicating that activin A acts on resting but not activated B cells [88]. Like TGFβ, activin A upregulates IgA synthesis in LPS-stimulated B cells by the induction of both Ig germline and post-switch α transcripts [89]. Finally, neutralization of activin A in immunized mice led to reduced circulating levels of IgE and a notable decrease in serum IL-4 levels [88]. 

Although B cells are able to produce Igs independently of T cells (i.e., great part of the gut IgA) [90], for the majority of antigens the activation of B lymphocytes requires the collaboration with a particular subpopulation of CD4^+^ T cells named follicular T helper cells (Tfh). TGFβ appears to control the differentiation of these cells, although its precise role is still very controversial [91]. Thus, by using mice with either T cell-specific loss or constitutive activation of TGFβ signaling, it has been reported that TGFβ is required for the maturation of Tregs, which induce Tfh apoptosis. Moreover, peripheral Tfh cells escaping TGFβ control were resistant to apoptosis [92]. Conversely, other authors show that TGFβ signaling induces the formation of antigen-specific Tfh cells in the context of influenza virus mouse infection [93]. TGFβ suppress the expression of CD25, IL-2 signaling, and mTOR activation on virus-specific Tfh precursors, promoting their differentiation [93]. Finally, two additional studies show that TGFβ and Activin A promote initial Tfh differentiation in humans, but not in murine CD4^+^ T cells [94,95]. 

Different subpopulations of Tregs have been involved in the control of B cell activation and antibody production. Among them induced Tregs suppress autoantibody secretion by B cells both in vitro and in vivo through the production of TGFβ [96]. A new subpopulation of Tregs with an important contribution to the regulation of humoral immune responses has recently been identified. These cells, called regulatory follicular T (Tfr) cells, migrate into the germinal center and inhibit Tfh-mediated B-cell activation and antibody production [97]. Mice with a deficient TGFβ and IL-2 signaling in T cells exhibit a reduced Tfr development, resulting in an increased Tfh differentiation, enhanced germinal center responses, and increased plasma cell infiltration [98]. In addition to Tfr cells, CD4^+^CD25^−^LAG3^+^ Treg cells suppress the differentiation of Tfh cells, GC development, and antibody production. These cells act by a mechanism dependent on TGFβ3 production, which modulates B cell activity through the inhibition of several important pathways such as STAT6 (Signal transducer and activator of transcription 6), Syk (A tyrosine-protein kinase), and NFκB (Nuclear factor κ B) p65 with the requirement of PD-1 (programmed cell death protein 1) expression on B cells. Moreover, these LAG3^+^ (Lymphocyte-activation gene) Tregs are capable of limiting lupus disease progression in Fas deficient MRL/lpr mice [99,100]. In turn, resting B cells, probably presenting self-antigens, control Treg homeostasis through TGFβ3 and so presumably remain defensive while preventing autoimmune responses [101].

These effects described for TGFβ superfamily members on B cell activation are supposed to take place in a paracrine manner, but the potential contribution of autocrine TGFβ to B cell function is still under investigation. It has been recently reported that TGFβ1 production by resting human B cells is negatively controlled by activation and it may contribute to excessive immune responses and autoimmunity [102]. Another recent study found that in vitro stimulated, but not resting, human B cells are able to release active TGFβ from surface GARP (glycoprotein A repetitions predominant)/latent TGFβ1, that can act in an autocrine manner to promote IgA class switching [103]. It is possible that in a normal anti-inflammatory environment, resting B cells produce TGFβ1, thus favoring tolerance, but when a pathogen insult occurs, B cells activate and deviate cytokine production to the pro-inflammatory profile, allowing immune response development. Moreover, the regulation of bioactive TGFβ1 availability from a B cell could rely not only on other nearby B cells, but also on its cognate cell partners. It was previously reported that IgA production in murine Peyer’s patches requires interaction of B cells with subepithelial dendritic cells expressing integrin αVβ8, which activates latent TGF-β produced by an undefined cellular source [104]. Likewise, it has recently been observed that human intestinal DCs and blood monocytes highly express integrin αvβ8 [105,106].

## 3. B Cell Tolerance and Autoimmunity

### 3.1. Mechanisms for B Cell-Mediated Autoimmunity and Tolerance

B cells express a highly diverse repertoire of antigen receptors that enable them to recognize any potential foreign antigen. This heterogeneity results from the stochastic genetic recombination in V(D)J gene segments at the heavy and light chain loci during B cell development in the bone marrow. The random nature of this rearrangement process entails the potential risk of generating B cell clones that recognize self-antigens and, therefore, are capable of promoting autoimmune reactions. These potentially autoreactive cells need to be either deleted or silenced in the bone marrow or in the periphery through a series of complex mechanisms grouped under the term “immunological tolerance”. Both B cell intrinsic and extrinsic mechanisms control these tolerance processes [107,108]. TGFβ superfamily members participate actively in the regulation of B cell differentiation, survival, and activation. B-TβRII-KO mice with a selective deficiency in TGFβ signaling in B cells show increased levels of serum Igs and produce anti-ds DNA antibodies [76], although these autoimmune manifestations are less severe than those observed in mice TGFβ1-KO or T-TβRII-KO (Tgfbr2^f/f^ Cd4-Cre) mice which develop severe multi-organ inflammation and autoimmunity [4,5,28]. Although the mechanisms accounting for the differences in the severity of the autoimmune syndrome observed in these mutant mice have not been characterized in detail, they may be related to the fact that TGFβ signaling controls immune homeostasis through multiple cell types (i.e., DCs, macrophages, NK (Natural Killer) cells, T cells) that can be affected in TGFβ1-KO mice and/or because T cells are master regulators of self-tolerance (Treg cells inhibit self-reactive responses and inflammation).

In addition to the above-mentioned processes, different subsets of specialized regulatory B cells control tolerance. The first definitive evidence for the existence of regulatory B cells (Bregs) came from the observation that mice deficient in B cells developed an exacerbated form of experimental autoimmune encephalomyelitis (EAE) in comparison to wild type controls [109]. The best characterized mechanism by which Bregs exert their regulatory functions is through the production of IL-10 [110]. However, Breg cells also control immune responses by IL-10-independent mechanisms such as the expression of ligands that induce apoptosis in target cells (FasL, TRAIL (TNF-related apoptosis-inducing ligand), or PD-1L (PD-1 Ligand)) [111] or by the production of TGFβ1 [112]. Thus, LPS-activated B cells express high levels of TGFβ1 and their transfer into prediabetic NOD (non-obese diabetic) mice inhibits spontaneous Th1 immunity and disease progression by down-regulating T cell autoimmunity or by modulating the function of APCs (Antigen Presenting Cell) [112]. These activated murine B cells also exert potent inhibitory effects on CD8^+^ T cells, inducing their anergy that is rescued by treatment with anti-TGFβ antibodies [113]. Bregs also express the TGFβ latency-associated peptide (LAP) and are able to promote graft survival by enhancing Treg expansion [114]. Moreover, resting splenic B cells can also expand Tregs, and this expansion requires TGFβ3 and signaling through the TCR (T-cell receptor) and CD28 [101]. Finally, studies in EAE show that B cell-derived TGFβ1 can constrain Th1/Th17 responses through the inhibition of APC activity. In this regard, mice with selective B cell-deficiency in TGFβ1 have a greater EAE susceptibility and increased myeloid DCs activation and Th1/Th17 responses in the CNS (Central Nervous System), thus suggesting that B cell-derived TGFβ1 can limit the induction of autoimmune neuroinflammation [115]. New evidence indicates that TGFβ1 together with IL-10 mediate inhibition of B cell responses by suppression of metabolism in murine and human B cells and ameliorate SLE in both induced and spontaneous murine models [116]. Furthermore, a potential role for B cell membrane-associated TGFβ1 has recently been evidenced in a chemically induced SLE model [117]. 

The suppressive effect of TGFβ produced by Breg cells was also demonstrated in humans. Human Bregs were able to enhance FoxP3 and CTLA-4 (cytotoxic T-lymphocyte-associated protein 4) expression in Tregs by TGFβ and cell-to-cell contact-dependent mechanisms [118]. Importantly, these TGFβ producing Bregs were significantly decreased in patients with rheumatoid arthritis, particularly in those with interstitial lung disease complications [119], whereas they expanded in response to allergen stimulation in tolerant but not in allergic subjects [120]. 

### 3.2. TGFβ Induced IgA and Mucosal Homeostasis

A large number of microorganisms, commonly referred to as microbiota, colonizes the skin and mucosae, particularly the gastrointestinal mucosa. Complex bidirectional interactions are stablished between the microbiota and the host immune system that ultimately determine the state of both compartments in terms of composition and immune activation [121,122]. TGFβ is an important participant in this network of interactions, especially in the gastrointestinal tract where this growth factor influences CD4^+^ T cells (regulating Treg and Th17 differentiation), innate lymphoid cells, B cells, phagocytes, and intestinal epithelial cells (IECs) [123]. IECs, phagocytes (some particular populations of DC and macrophages) receiving stimuli from the microbiota and apoptotic IECs and different populations of CD4^+^ T cells, are a major source of bioactive TGFβ [124,125,126]. Furthermore, these interactions support the basis for the construction of physical and chemical protective barriers at the mucosal surfaces. Disruptions in the integrity of the gut barrier may result in the development of aberrant immune responses against microbial antigens that could promote either local and/or systemic inflammatory/autoimmune diseases [127,128,129].

IgA produced by B cells and translocated into the lumen is an essential component of the gut barrier. It is secreted in high amounts daily and acts by neutralizing viruses and pathogenic bacteria (high-affinity IgAs) while restricting commensal bacterial penetration (low-affinity IgAs). In this way, IgA contributes to the maintenance of mucosal tolerance [130]. TGFβ plays a crucial role in the generation of IgA class-switched B cells in the inductive sites of the gut-associated lymphoid tissues (GALT), the Peyer’s patches, the isolated lymphoid follicles, and mesenteric lymph nodes. In this regard, loss or a significant reduction in IgA secretion was found in mice harboring B cells with deficiency of either TβRII [76], Smad-2 [84], Smad-3, or Smad-4 [131]. Conversely, limited Smad-7 activity in B cells results in increased IgA production [83]. The molecular mechanism that links TGFβ to IgA production is the docking of Smad-2/-3/-4 complexes onto the regulatory sequence of the Ig Cα gene, which, besides multiple co-factors (including PU.1 and RUNX3), promote germ-line transcriptions and switch recombination [132,133]. IgA production in the GALT occurs through T cell-dependent and independent pathways and TGFβ is involved in both pathways [134]. In the gut tolerogenic environment, retinoic acid secreted by IECs promotes the development of CD103^+^ DCs, producing high amounts of anti-inflammatory factors such as TGFβ, IL-10, and retinoic acid [135,136]. These factors promote the generation of Tregs that in turn produce high amounts of TGFβ and IL-10 [124,137]. In addition, Tregs may further differentiate into FoxP3^+^ Tfh cells that drive the generation of T-dependent IgA class-switched B cells in the inductive sites of the GALT [138]. More recently, it has been described that in Peyer’s patches, Th17 cells can acquire a Tfh phenotype inducing IgA production [139]. Lamina propria-resident CD103^+^ DCs may also deliver antigens and TGFβ to B cells and initiate T-independent IgA production in this location [140,141]. 

## 4. Conclusions and Future Perspectives

Accumulating evidence points to TGFβ superfamily members as important regulators of B cell responses at multiple levels; from their development in the bone marrow up to their activation and final differentiation into antibody secreting plasma cells in the periphery. TGFβ superfamily members act in either an autocrine or paracrine fashion and modulate immune tolerance and homeostasis (Figure 2). However, further investigations are needed to clarify the cellular source of these factors, the precise activities of each superfamily member, and the immune cell and the context (homeostasis vs inflammation) where these factors act during the development of normal and pathological humoral immune responses. These studies are essential for the development of new selective TGFβ targeted therapies that either promote or inhibit desirable or deleterious B cell activities, respectively. Since the TGFβ superfamily members possess highly pleiotropic activities in almost every organ and tissue, non-selective approaches can result in severe side effects [142]. Thus, the potential risks of using either neutralizing TGFβ antibodies or TGFβ analogs in humans must be seriously considered. 

Additional approaches could focus on molecules that regulate the strength of TGFβ signaling. One promising candidate is BAMBI (BMP and Activin Membrane-Bound Inhibitor), a transmembrane protein structurally similar to TβRI but lacking the kinase domain required for signaling. It antagonizes TGFβ superfamily signals by preventing the formation of active receptor complexes upon ligand binding [143]. Recently, it has been evidenced that BAMBI forms part of a rheostat-like machinery that fixes the appropriate intensity level of TGFβ signaling in CD4^+^ T cells. Through the regulation of CD25 expression and the intensity of IL-2 signaling, BAMBI positively and negatively controls the differentiation of Th17 and Treg cells, respectively, and consequently the development of autoimmune diseases in rodents [144]. The role of BAMBI in the regulation of humoral B cell responses is currently under investigation.

## Figures and Tables

**Table 1:** Main components of the TGFβ superfamily signaling canonical pathway.

Ligand	Type I Receptor	Type II Receptor	R-Smad	Co-Smad	I-Smad
TGFβ1, TGFβ2, TGFβ3	ALK-5	TβRII	Smad-2, -3	Smad-4	Smad-7
BMP-2 to 7, BMP-8A, BMP-8B, BMP-9, BMP-10	ALK-1, -2, -3, -6	BMPRII/IIB ActRII/IIB	Smad-1, -5, -8	Smad-4	Smad-6, -7
Activins	ALK-4 (ActRIB), ALK-7	ActRII/IIB	Smad-2, -3	Smad-4	Smad-7
GDFs	ALK-2, -3, -6	BMPRII, ActRIIA/B	Smad-1, -5, -8	Smad-4	Smad-6, -7
	ALK-4, -5, -7		Smad-2, -3		
Nodal	ALK-4, -7	ActRII/IIB	Smad-2, -3	Smad-4	Smad-7
AMH	ALK-2, -3, -6	AMHRII	Smad-1, -5, -8	Smad-4	Smad-6, -7

ALK: Activin receptor-like kinase.
